# Identification of Cross-Country Skiing Movement Patterns Using Micro-Sensors

**DOI:** 10.3390/s120405047

**Published:** 2012-04-18

**Authors:** Finn Marsland, Keith Lyons, Judith Anson, Gordon Waddington, Colin Macintosh, Dale Chapman

**Affiliations:** 1 University of Canberra, ACT 2601, Australia; E-Mails: Keith.Lyons@canberra.edu.au (K.L.); Judith.Anson@canberra.edu.au (J.A.); Gordon.Waddington@canberra.edu.au (G.W.); Colin.Macintosh@ausport.gov.au (C.M.); Dale.Chapman@ausport.gov.au (D.C.); 2 Australian Institute of Sport, Leverrier Crescent, Bruce, ACT 2617, Australia

**Keywords:** accelerometer, gyroscope, kinematics, technique analysis

## Abstract

This study investigated the potential of micro-sensors for use in the identification of the main movement patterns used in cross-country skiing. Data were collected from four elite international and four Australian athletes in Europe and in Australia using a MinimaxX™ unit containing accelerometer, gyroscope and GPS sensors. Athletes performed four skating techniques and three classical techniques on snow at moderate velocity. Data from a single micro-sensor unit positioned in the centre of the upper back was sufficient to visually identify cyclical movement patterns for each technique. The general patterns for each technique were identified clearly across all athletes while at the same time distinctive characteristics for individual athletes were observed. Differences in speed, snow condition and gradient of terrain were not controlled in this study and these factors could have an effect on the data patterns. Development of algorithms to process the micro-sensor data into kinematic measurements would provide coaches and scientists with a valuable performance analysis tool. Further research is needed to develop such algorithms and to determine whether the patterns are consistent across a range of different speeds, snow conditions and terrain, and for skiers of differing ability.

## 1. Introduction

Cross-country skiing uses a range of skiing techniques to cover various types of terrain efficiently. No other sport has such a wide range of repetitive movement patterns used in one event. There are two main event styles, skating and classical, each of which has four main sub-techniques or “gears” used in competition [1]. Efficient selection of an appropriate gear depends on the gradient of the terrain and the velocity at which a skier is travelling [2]. The frequent changing of gears during competition complicates any attempt to quantify how effective movements are. Andersson *et al.* [2] reported an average of 29.1 gear changes during a 1.43 km skating time trial. It is unlikely that any two athletes will ski a race course using the exact same techniques in the same places.

A number of recent cross-country skiing studies have measured combinations of ski speeds, cycle rates, cycle lengths and the distribution of ski technique use during competition, simulated competition and using ski-simulation activities in the laboratory [2–7]. These studies have shown that ski speed in different terrain varies with cycle rates, cycle lengths and technique selection, and that these kinematic data are useful in analysing ski performance. Andersson *et al.* [2] noted that the self-selection of technique is related to performance capacity; better ranked skiers use a higher proportion of higher gears. Similarly, Sandbakk *et al.* [4] reinforced Smith's [8] observation that faster skiers generally have greater cycle lengths.

In the field these kinematics have generally been measured using lapsed-time video analysis. This is a time intensive method and results in considerable delay in the sharing of data with coaches and athletes, and is generally impractical during competition. Sandbakk *et al.* [4] used 10 video cameras to cover a 1.82 km time trial, while Andersson *et al.* [2] filmed athletes from behind while following on a snowmobile. Technology that enabled quick measurement of kinematics in the field would provide sport scientists, coaches and athletes with a valuable tool for performance analysis, enabling them to evaluate improvements in power output or skiing efficiency through changes in kinematics.

Preliminary work with combinations of micro-sensors indicates that they have the potential to identify and measure cross-country skiing kinematics [9]. Micro-sensors have been used for performance analysis in a number of sports, including Australian rules football [10], rugby [11], soccer [12], swimming [13], kayaking [14] and snowboarding [15]. Fulton *et al.* [16] used inertial sensors to quantify kick-count and kick-rates in swimming through the use of algorithms identifying each movement cycle. Similarly, Harding *et al.* [15] used inertial sensors to classify different aerial aerobatics in snowboarding, and Janssen and Sachlikidis [14] used inertial sensors and GPS to measure stroke rates, velocity and accelerations in kayaking. In the team sports mentioned above accelerometers and GPS have been used to quantify performance loads, measuring distances travelled and the number and intensity of accelerations during matches [10–12]. In all of these studies the micro-sensor units are relatively unobtrusive and can be used in normal training sessions, and in some instances in competition. Recently Myklebust *et al.* [9] used data from five accelerometers located around a skier's body and equipment to identify technique cycles and measure cycle rates. Algorithms were developed to process the accelerometer data and detect when poles and skis came into contact with the snow. Although this methodology allows for a robust technique analysis, the number of units and total weight of equipment used appears prohibitive for use in the daily training environment or during competition.

The aim of the current study was to determine whether a single micro-sensor unit attached to the body could be used to identify effectively each of the main techniques used during cross-country skiing competition. For such identification to be useful, the distinctive data patterns for each technique should be recognisable regardless of factors such as snow conditions, equipment used, skier speed, gradient of the terrain, standard of the skier or athlete fatigue. Successful identification of a cycle pattern for each technique would be the first step towards developing algorithms for automatic detection of cross-country skiing kinematics.

## 2. Experimental Section

### 2.1. Participants

Two groups of participants were used: an international group (IG) and a national Australian group (AG). IG athletes (three male, one female) had International Ski Federation (FIS) ranking points between 2.0 and 27.0 on the first ranking list for 2011/2012. Each athlete in this group had achieved at least one podium result in FIS World Cup competition between 2009 and 2011. AG athletes (three male, one female) were all members of the Australian Cross-Country Ski Team and had FIS ranking points between 60.0 and 97.0 on the first ranking list for 2011/2012. All FIS World Cup results and rankings are available from http://www.fis-ski.com. Data were collected at Davos in Switzerland, Beitostoelen in Norway, and at Falls Creek in Australia.

Ethics approval for the study was granted by the Australian Institute of Sport Ethics Committee (approval number 20102002) and the University of Canberra Committee for Ethics in Human Research (approval number 10-146). All participants were supplied with a participant information sheet prior to testing and given the opportunity to ask additional questions before signing written consent forms.

### 2.2. Equipment

**Figure 1.**
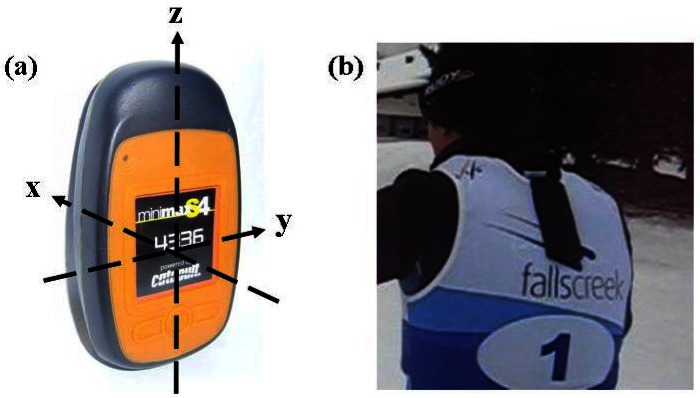
(**a**) Micro-sensor unit; (**b**) Modified bib with micro-sensor in pouch.

Data were collected using a commercially available micro-sensor unit (MinimaxX™ S4, Catapult Innovations, Melbourne, Australia). The micro-sensor unit contained a triaxial accelerometer (100 Hz, ±6 g), a gyroscope (100 Hz, ±1,000 d/s), a Global Positioning System (GPS) device (Fastrax, 5 Hz) and a magnetometer (30 Hz). The unit has dimensions of 2.0 × 4.8 × 8.5 cm and weighs approximately 67 g. The reliability of the MinimaxX for sport analysis was previously demonstrated by Boyd *et al.* [10]. The validity of the distance covered at speeds of <2–5 m/s ranges between 1.7–3.8% with a reliability of 1.2–2.6% [17]. Cross-country skiing specific reliability was confirmed using two units worn concurrently by an athlete while rollerskiing. Calibration of the accelerometer and gyroscope was performed using the same method as described by Harding *et al.* [15]. The positioning configuration of the accelerometer is shown in Figure 1(a) and described in more detail in Section 2.5. Video data were collected using a Sony Handycam HDR CX110 (60 Hz) and a Canon IXY 900 (30 Hz).

Each athlete wore a micro-sensor located in a carrying pouch sewn onto the back of a standard competition race bib used in international cross-country skiing events. The pouch positioned the micro-sensor in the centre of the upper back approximately 5 cm lower than the base of the neck, which is approximately the same position as occurs with the standard harness supplied for use with the micro-sensor. This modified bib was worn by the participants over the top of their training clothing.

### 2.3. Description of Techniques

The following cross-country skiing techniques were chosen for analysis:

Skating techniques: (classified as gears G2-G5 according to Nilsson *et al.* [1])
  - Offset Skate (G2)
  - Double Time (G3)
  - Single Time (G4)
  - Free Skate (G5)
Classical techniques: (universally used definitions)
  - Double Pole (DP)
  - Kick Double Pole (KDP)
  - Diagonal Stride (DS)

**Figure 2.**
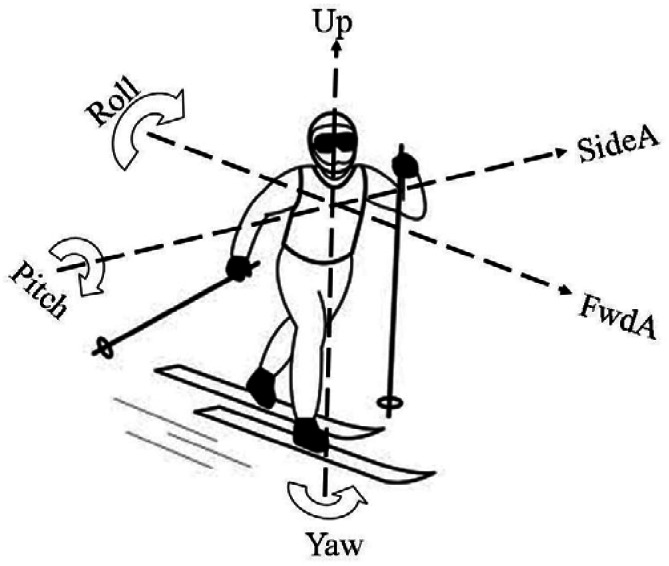
Orientation of accelerometer and gyroscope relative to the skier.

The following terminology is used to describe the accelerometer and gyroscope signals (Figure 2):

FwdA = forward/backward acceleration along the x-axis of the micro-sensor unit
SideA = left/right acceleration along the y-axis of the micro-sensor unit
UpA = up/down acceleration along the z-axis of the micro-sensor unit
Roll = angular acceleration about the x-axis
Pitch = angular acceleration about the y-axis
Yaw = angular acceleration about the z-axis

### 2.4. Data Collection

Participants were videoed by a stationary camera from side-on performing each ski technique while wearing the modified bib containing the micro-sensor unit. Each technique was performed at a moderate pace for 20–30 seconds on a straight section of track. At the beginning of each data collection session a synchronisation marker point was set by tapping the micro-sensor unit sharply three times. Video footage was recorded on a single hand-held video camera continuously from the marker point through to the end of each data collection session. The total time taken for one session of on-snow data collection was 3–4 minutes per athlete. The terrain chosen for data collection included flat and moderate uphill sections appropriate for use of the different techniques. Participants were instructed to ski at a “moderate intensity slightly faster than their normal easy distance skiing pace”.

Data collection took place on groomed ski tracks. In general the snow was well packed and the conditions were moderately fast, otherwise there was no standardisation of the snow crystal type, temperature or humidity. There was no standardisation of participants' ski equipment or equipment preparation, with participants free to use their own skis, boots and poles.

### 2.5. Data Processing and Interpretation

Data files from the micro-sensors and corresponding video files were downloaded to a portable computer (Acer Aspire) and uploaded to software packages designed for use with the MinimaxX units (Logan V41.0, Australian Institute of Sport, Canberra, Australia; and Logan Plus 4.5.0, Catapult Innovations, Melbourne, Australia). The software enabled easy viewing of the accelerometer and gyroscope data plotted against time. The gyroscope and accelerometer data from two subjects were initially filtered with a low-pass Butterworth filter using cut-off frequencies in the range 1–4 Hz. Following visual inspection of the resultant filtered data a cut-off frequency of 2.0 Hz for all the accelerometer data and 1.0 Hz for all the gyroscope data was considered acceptable. The choice of these frequencies enabled the major features of each technique to be clearly identified while allowing minor differences between individuals to remain visible. The data and video files were synchronised manually by matching up the visual indicators of the initial marker point. Ten second blocks of each technique were identified and marked for analysis.

When viewing the gyroscope data it was decided to use a different scale for different dimensions; Pitch is presented in the range −150 to +150 d/s, while Roll and Yaw are presented in the range −75 to +75 d/s. These relative scales were chosen due the magnitude of Pitch generally being substantially larger than the other two dimensions for most of the cross-country skiing techniques.

Technical interpretation of the data was performed by a cross-country skiing coach (Author F.M.) with over 20 years of experience of international competition and working with athletes on technique.

When analysing and interpreting accelerometer data it is important to be aware of the effect of gravity. When the micro-sensor is stationary the acceleration along each axis ranges from −1.0 g to +1.0 g, depending on unit orientation. The MinimaxX was configured so that FwdA has a value of +1.0 g when the Z axis is in a vertical position as shown in Figure 1(a). In this position SideA and UpA have values of 0.0 g. When the MinimaxX unit is rotated forward so that the X-axis is vertical, then UpA has a value of 1.0 g and FwdA and SideA have values of 0.0 g. The accelerometer data presented in this paper is a combination of movement-induced accelerations through the micro-sensor unit and gravity acceleration due to the orientation of the unit. While it is possible to correct for gravity using algorithms [18], as the main aim of this study was to identify the basic technique patterns this was not considered necessary.

## 3. Results and Discussion

### 3.1. Characteristics of Each Technique

**Figure 3.**
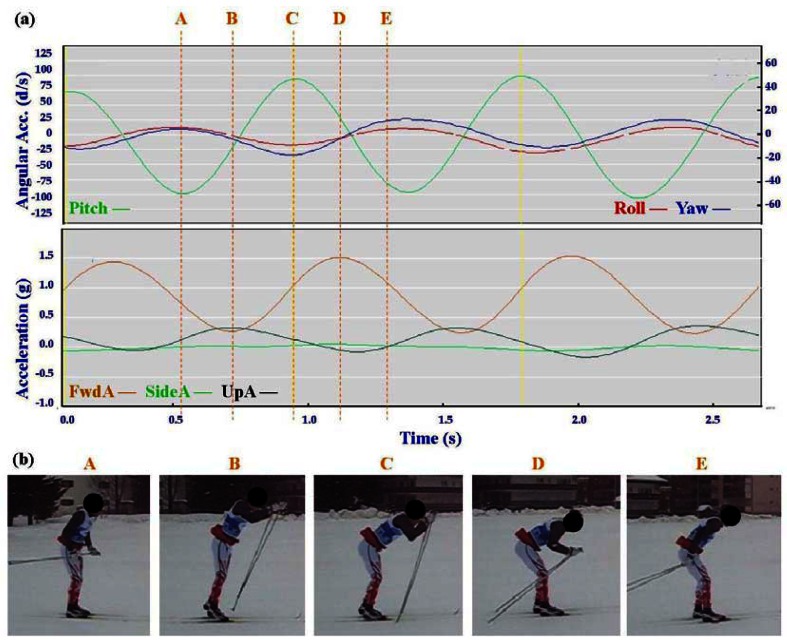
(**a**) Screenshot of Double Poling (DP) gyroscope and accelerometer traces against time; (**b**) DP body position for selection points.

**Figure 4.**
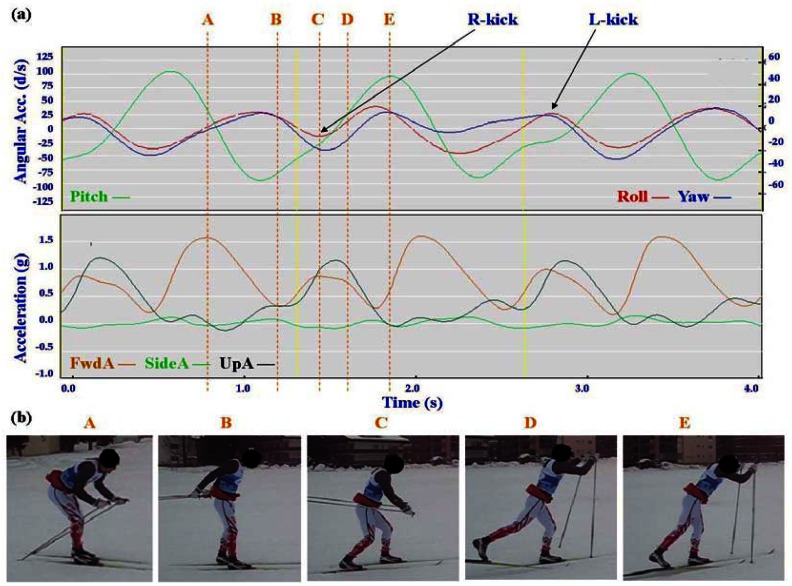
(**a**) Screenshot of Kick Double Poling (KDP) gyroscope and accelerometer traces against time; (**b**) KDP body position for selection points.

**Figure 5.**
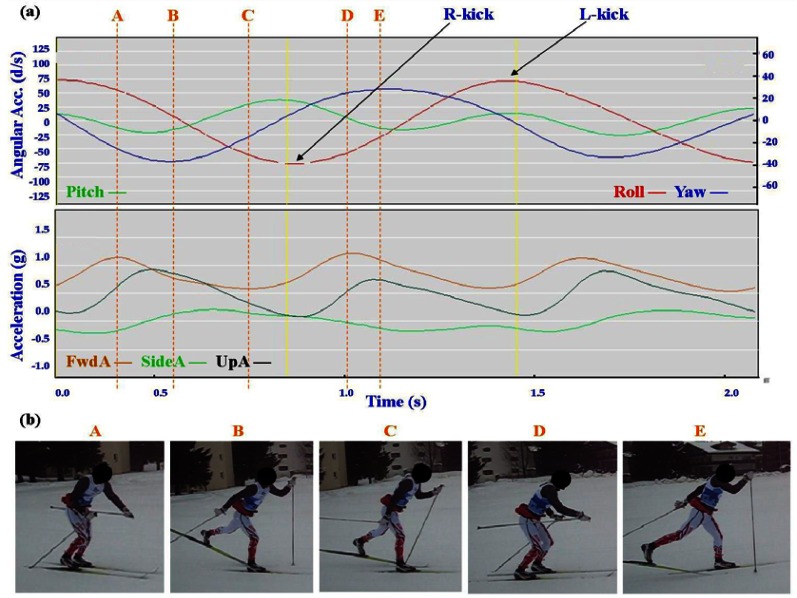
(**a**) Screenshot of Diagonal Stride (DS) gyroscope and accelerometer traces against time; (**b**) DS body position for selection points.

**Figure 6.**
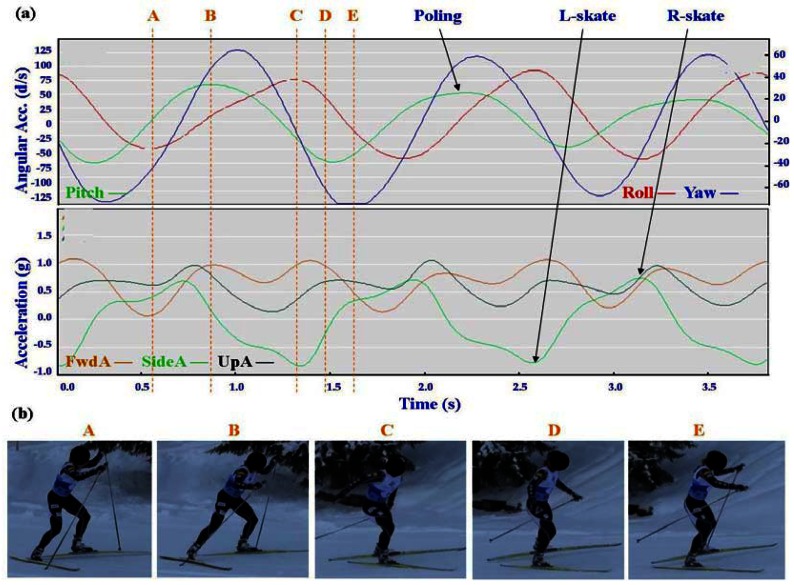
(**a**) Screenshot of Offset Skate (G2) gyroscope and accelerometer traces against time; (**b**) G2 body position for selection points.

**Figure 7.**
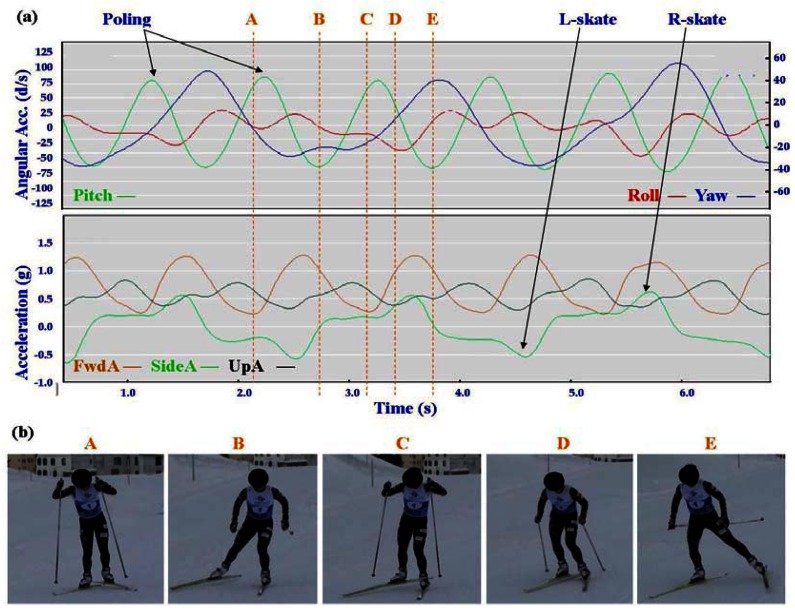
(**a**) Screenshot of Double Time (G3) gyroscope and accelerometer traces against time; (**b**) G3 body position for selection points.

**Figure 8.**
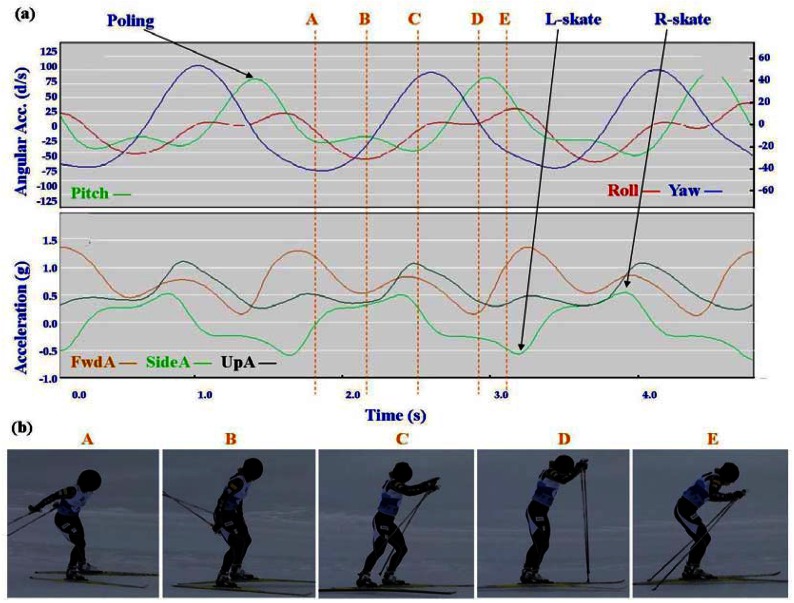
(**a**) Screenshot of Single Time (G4) gyroscope and accelerometer traces against time; (**b**) G4 body position for selection points.

**Figure 9.**
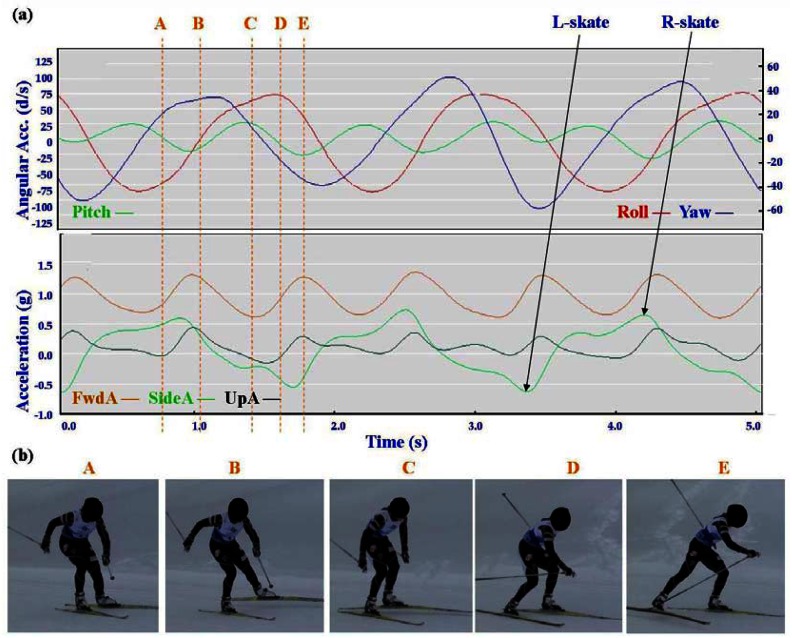
(**a**) Screenshot of Free Skate (G5) gyroscope and accelerometer traces against time; (**b**) G5 body position for selection points.

Typical signal patterns created by the micro-sensors for each technique are shown below in Figures 3–9.

Each figure includes two components: (a) a screen-shot of the gyroscope and accelerometer data curves for one athlete; and (b) images A-E identifying corresponding positions in the cycle of each technique. One IG athlete is used to demonstrate the classical techniques (DP, KDP and DS) and a different IG athlete is used for the skating techniques (G2-5).

#### 3.1.1. General Observations

The micro-sensor data collected demonstrated a clear repetitive cycle for each of the techniques examined. The general characteristics of the accelerometer and gyroscope data common to all athletes for each technique are summarised in Table 1 and Table 2 below.

In all techniques involving poling (DP, KDP, DS, G2, G3, G4) the peak of the Pitch precedes the main peak of the FwdA, or in the case of G2 the double peak of FwdA. Visually from the video data the peak Pitch corresponds to a point in the middle of the poling action. In the case of G5 which does not involve poling the peak pitch is considerably lower than for the other techniques. For all techniques there are some minor differences in timing and features between athletes, these are described in more detail under Section 3.2.

In Figures 4–9 left and right labels demonstrate that movements on one side of the body can be distinguished from the other. For the Classical techniques L-kick and R-kick are defined by a peak/trough in the Yaw curve as the leg is kicking backwards, which results in the rotation of the upper body. For Skating techniques L-skate and R-skate are identified by the distinct peaks/troughs in SideA.

#### 3.1.2. Classical Techniques

The three classical techniques are relatively easy to distinguish using either the accelerometer data or the gyroscope data. SideA is minimal for all three techniques, understandably so as the skis generally remain parallel during classical skiing and only move sideways during turning techniques (which are not covered in this paper). The timing and magnitude of FwdA and UpA identify the different techniques, as shown in Table 1 above. From the gyroscope data DS can be identified by the relatively smaller magnitude of Pitch compared to Roll and Yaw; DP is characterised by the very rhythmic Pitch and minimal Roll and Yaw; while KDP is identified by the slightly asymmetric Pitch curve. When the gyroscope data are examined using a filter of 2 Hz instead of 1 Hz this separates into a minor peak and a major peak (not shown). Left and right kicks during the KDP and DS are shown by the Roll peaks and troughs. For DS the full extension of the leg/arm drive is indicated by the Yaw peaks and troughs.

#### 3.1.3. Skating Techniques

The most distinctive common features of the skating techniques are the consistent asymmetric SideA peaks and troughs of moderate magnitude, which are indicative of the left and right leg skating motions. What distinguishes the different techniques is the timing of the poling action relative to the skating action.

The two most similar techniques are G2 and G4, which are used in very different terrain (G2 in steeper uphills and G4 on flatter, faster terrain). Both techniques feature one poling action to two skating actions (left/right); the main difference between these gears is in the timing of poling. In G4 the peak Yaw precedes the peak Pitch, while in G2 the peak Yaw is just slightly after the peak Pitch. This is consistent with visual observation of the two techniques, where the pole plant of G2 corresponds closely with the planting of the ski on the leading side, and the pole plant of G4 precedes a push onto the ski. From the perspective of FwdA, G4 has a clear major and minor peak, while G2 has more of a double peak. In G4 the higher peak of FwdA comes at the end of the poling motion; the smaller peak corresponds to the skate without poling. The smaller UpA peak matches up with the minor FwdA peak of G4, while for G2 the peak of UpA precedes the double peak. Another factor to note with G4 is that the Yaw trough is wider than the Yaw peak. This indicates that the return of the poles is faster than the poling action.

G3 and G5 techniques are identified more easily, by the double frequency of poling action in G3 (indicated by the Pitch) and the absence of poling in G5 (indicated by reduced magnitude of Pitch).

### 3.2. Comparison between Athletes

**Figure 10.**
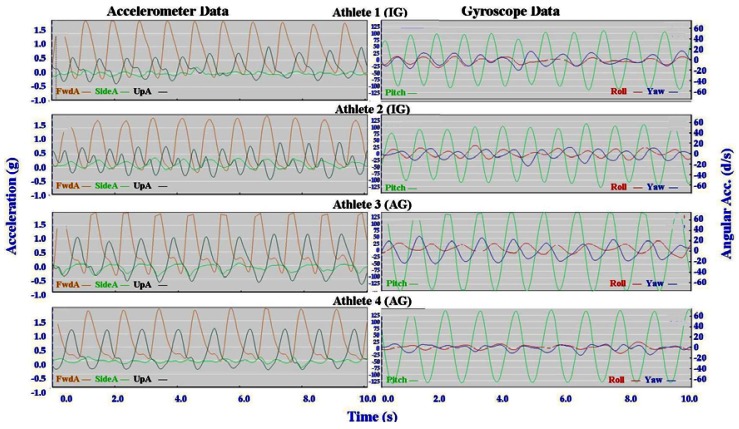
Comparison of Double Pole (DP) gyroscope and accelerometer traces for four athletes (two from the IG and two from the AG).

**Figure 11.**
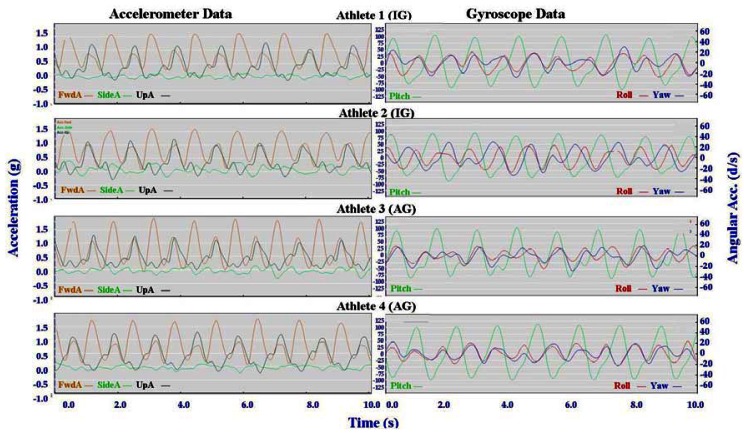
Comparison of Kick Double Pole (KDP) gyroscope and accelerometer traces for four athletes (two from the IG and two from the AG).

**Figure 12.**
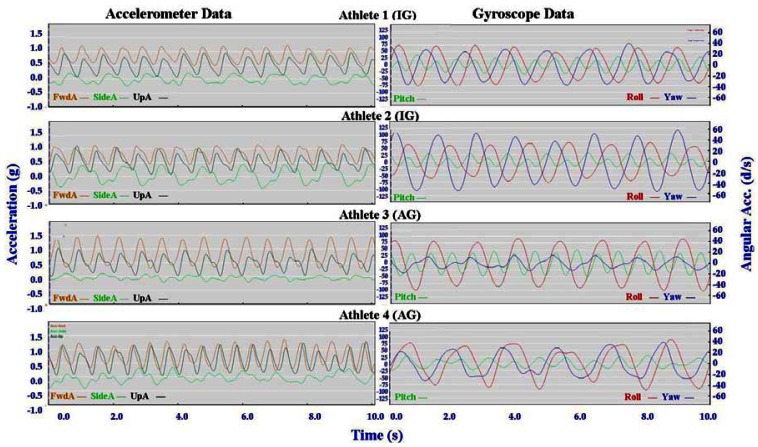
Comparison of Diagonal Stride (DS) gyroscope and accelerometer traces for four athletes (two from the IG and two from the AG).

**Figure 13.**
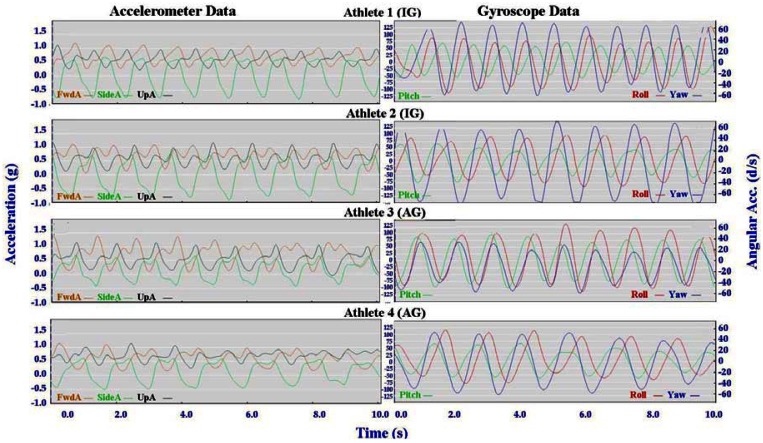
Comparison of Offset Skate (G2) gyroscope and accelerometer traces for four athletes (two from the IG and two from the AG).

**Figure 14.**
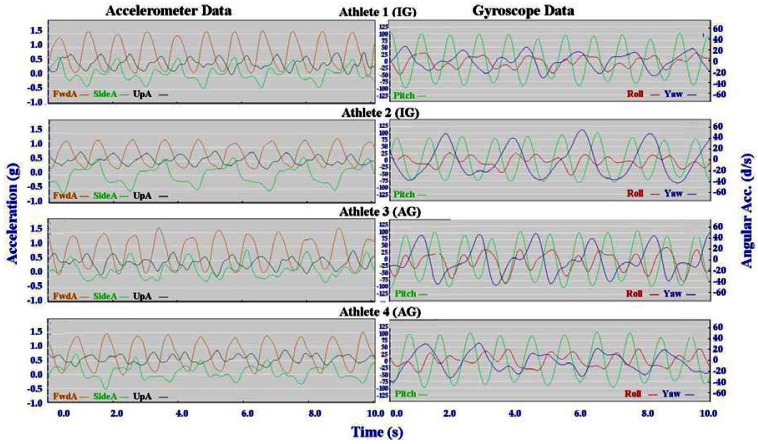
Comparison of Double Time (G3) gyroscope and accelerometer traces for four athletes (two from the IG and two from the AG).

**Figure 15.**
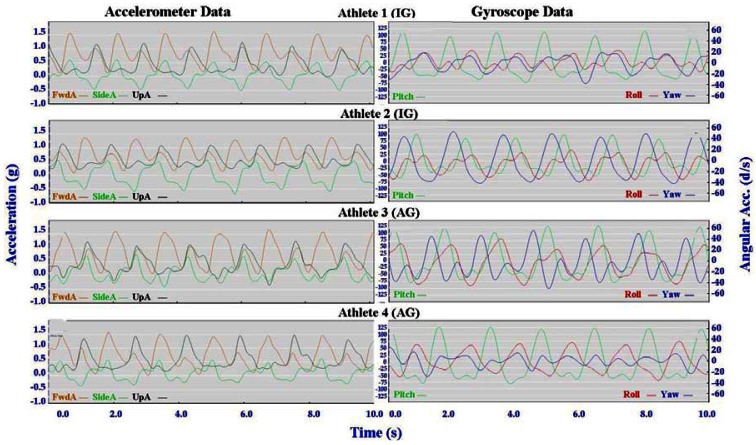
Comparison of Single Time (G4) gyroscope and accelerometer traces for four athletes (two from the IG and two from the AG).

**Figure 16.**
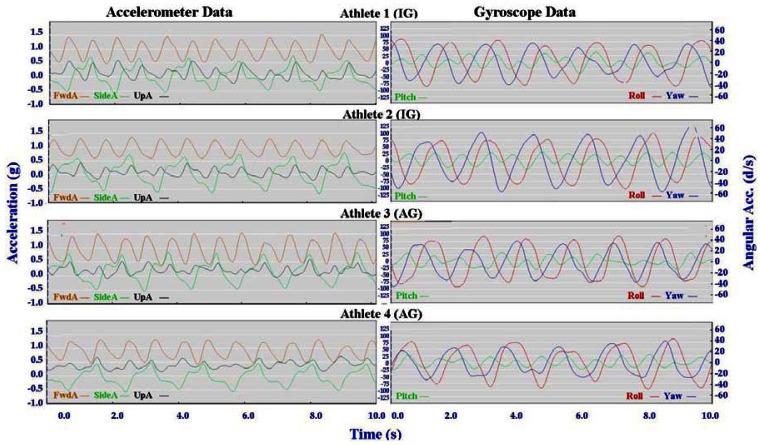
Comparison of Free Skate (G5) gyroscope and accelerometer traces for four athletes (two from the IG and two from the AG).

Individual accelerometer and gyroscope data patterns for four athletes are presented below in Figures 10–16 in order to demonstrate the similarities and differences for each technique.

#### 3.2.1. General Observations

The main features of the accelerometer signal patterns for each athlete have a number of similarities. The basic shape of the curves and the timing of accelerations are consistent, with small differences in the relative magnitudes and the smoothness of the curves distinguishing each athlete. In some instances a slight bump in a trace for one athlete appears as a double peak for another athlete; as noted in Section 3.1 above, this characteristic is affected by different levels of signal filtering. For the gyroscope traces the differences between athletes are more pronounced with some of the techniques. Though the general patterns can still be identified, the relative timing of angular acceleration in each dimension can vary noticeably and the relative differences in magnitude are more obvious.

No general differences between IG and AG athletes were detected. Fine differences between athletes within one group were as noticeable as differences between groups. It should also be noted that some differences could be attributable to different factors that were not controlled in this study, such as the gradient of the terrain, snow conditions and ski speed. The effect of gradient is discussed in Section 3.3.

#### 3.2.2. Classical Techniques

Figures 10–12 depict the classical techniques. For all three gears the slight differences in UpA peaks for different athletes were not readily apparent when viewing the video data. Similarly, the SideA visible for several athletes when performing DP and KDP could not be seen on the video. However for DS when a larger SideA was accompanied by a large peak in Yaw (as exhibited by Athlete 2 in Figure 12), this movement was manifest on the video footage in the form of increased upper body rotation. Conversely, the lower magnitude of Yaw exhibited by Athlete 3 in Figure 12 could be seen by a relatively stiff upper body with less arm drive.

**Figure 17.**
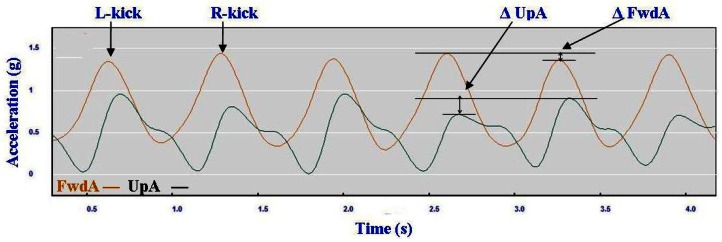
Differences between left and right kick in Diagonal Stride (DS).

With one of the athletes the use of micro-sensors detected subtle changes not previously noted. Figure 17 demonstrates lower FwdA and higher UpA corresponding to the kick from the left leg relative to the right. Subsequent inspection of the video footage confirmed this slight asymmetry and apparent one-sided inefficiency. This highlights the potential usefulness of micro-sensors for refining technique.

#### 3.2.3. Skating Techniques

The skating techniques are shown in Figures 13–16. For G2 there are notable differences in the appearance of the angular acceleration data for different athletes. With Athlete 3 in Figure 13 the curves for Pitch, Roll and Yaw are almost concurrent, while for Athlete 2 the curves have spread out slightly and the Yaw is of a higher magnitude. At first examination it appears that Athlete 1 has completely different timing, however this is because G2 is an asymmetric technique and Athlete 1 is the only athlete leading to the left (which causes the Yaw and Roll curves to be inverted). The two most similar traces are those of Athletes 2 and 4, one of whom is from the IG and one of whom is from the AG. Video footage shows that the timing of poling relative to skating on the leading side is similar for athletes 1, 2 and 4, while Athlete 3 plants their poles slightly earlier relative to the full commitment of weight to the skating ski. It is less clear as to which method is more efficient as gradients and athlete velocities were not controlled.

With the G3 and G4 gears the SideA data for Athlete 3 show a more irregular curve from cycle to cycle. Left and right skating movements can be identified, but the curves vary noticeably from skate to skate. This can be seen also with FwdA for Athletes 3 and 4 when using G3 and to a lesser extent G5. Potentially this could have significance relative to stability or technical efficiency, however, examination of the video data is inconclusive in this respect.

For G5 the SideA is very consistent for all athletes, as is the timing of the gyroscope data. The only minor difference in the gyroscope data for G5 is in the magnitude of Yaw, as demonstrated in the contrast between Athletes 2 and 3 in Figure 16. Incidentally, Athlete 2 has higher peaks of Yaw than the other athletes throughout all four skating gears, indicating increased upper body rotation.

### 3.3. Longitudinal Analysis

**Figure 18.**
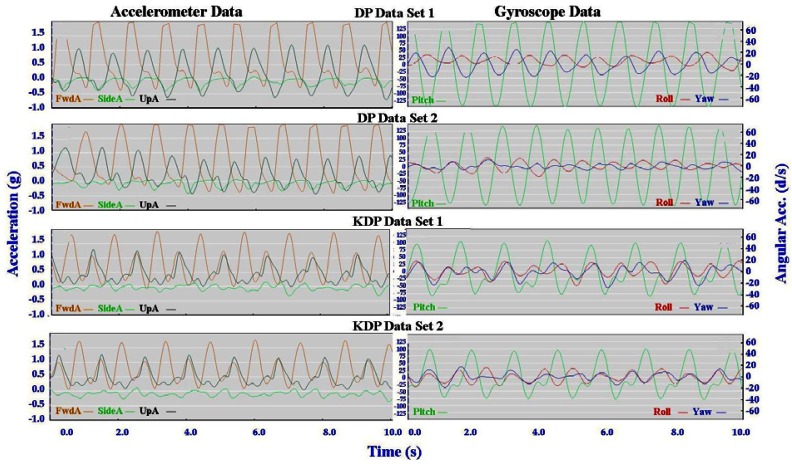
Longitudinal analysis of DP and KDP.

Longitudinal analysis of AG athletes at different locations underlined the need for further research. The two different data sets in Figure 18 were collected from the same athlete in different countries more than two months apart.

**Figure 19.**
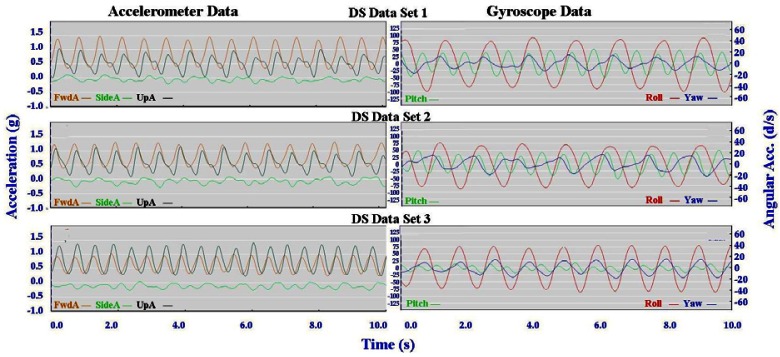
Longitudinal analysis of DS.

The similarities between the KDP accelerometer curves and the distinctive shape of the SideA curve in the DP data identify the athlete in Figure 18 as Athlete 3 in Figure 10. However, these similarities are not as clear when examining the DS data in Figure 19 below.

The technique is clearly DS, however the relative magnitudes of FwdA to UpA and Pitch to Roll are quite different. An explanation for these differences appears to be found in the different gradients in terrain used during data collection. While DP and KDP were performed on relatively flat tracks or slight inclines at the different locations, the hills used for DS were of varying gradients. Data Set 2 was collected on a slightly steeper slope than for Data Set 1, and Data Set 3 was collected on a much steeper hill. The accelerometer data observations at these different gradients seem logical; FwdA decreases and UpA increases as the incline becomes steeper. It is apparent that the micro-sensor traces are sensitive enough to detect the subtle changes in technique induced by the changes in gradient. However additional data from a range of athletes skiing at different gradients will be needed in order to examine this properly. As the slope becomes even steeper the Herringbone technique, not covered in this study due to the lack of appropriate terrain available at the testing locations, will also need characterisation.

### 3.4. Future Directions

The general features of the cyclical movement patterns for each technique (Tables 1 and 2) appear robust and should provide a good basis for the development of algorithms to classify these cycles. To the naked eye the combination of the three filtered accelerometer curves for each technique are distinctive, and the peaks of the Pitch curves assist in identifying poling frequency. However, further research is necessary to develop and validate any algorithms developed. This validation process would need to consider factors such as variation in terrain and snow conditions, while incorporating data collection at competition speeds and from a larger cross-section of athletes. It needs to be noted that the moderate speeds used in this study were chosen in order to minimise the impact on participants' training schedules, and the kinematics of athletes moving at race intensity would be of more interest to coaches. If technique cycles can be automatically detected then the next logical step would be to combine the inertial sensor data with GPS data to calculate cross-country skiing kinematics at different positions along a track or around a race course.

Future micro-sensor classification of technique should also include gears G6 (cornering techniques) and G7 (tucking without poling or skating), as defined by Andersson [2], and Herringbone (for steep uphills in Classical technique) as indicated in Section 3.3. Other variations of skating technique such as “jump skate” (a high speed version of G2) and “double-push skate” [19] should also be considered.

The subtle differences between athletes and also potential detection of left/right side asymmetry, as described in Section 3.2, seem to indicate that analysis of micro-sensor data also has potential to assist in fine tuning technique. Although the influence of the variables indicated above are significant, if these factors can be accounted for then micro-sensor data could also be used to characterise superior/inferior technique.

## 4. Conclusions

A single micro-sensor unit mounted on an athlete's back can be used to identify the cyclical movement patterns of the major ski techniques used in cross-country skiing. A combination of inertial sensor data enables the poling action to be identified clearly, as well as the skating and kicking actions on each side of the body. Cyclical movement patterns for each technique can be identified across a range of athletes of different ability at the same time as individual characteristics are observed. A more detailed analysis of micro-sensor data could potentially be used to refine technique; however before individual differences can be examined further it will be necessary to control the influence of variables including speed, snow condition and gradient of terrain. These factors also need to be taken into consideration in the development of algorithms to detect and classify the technique cycles.

This successful identification of technique cycles and suitability for algorithm development suggests that micro-sensors have potential to be used as a tool for performance analysis in cross-country skiing training and competition.

## Figures and Tables

**Table 1.** Classical technique features common to all athletes.

**Technique**	**FwdA**	**SideA**	**UpA**	**Roll**	**Pitch**	**Yaw**
DP	1 major peak each cycle (poling)	minimal; cycle visible for some athletes only	1 small peak each cycle, coinciding with FwdA troughs	small but visible peaks <20 d/s each cycle	peak > 75d/s identifies poling	small but visible peaks <20 d/s each cycle
KDP	1 minor peak each cycle (kick); 1 major peak each cycle (poling)	minimal	1 peak each cycle, coinciding with minor FwdA peak	identifies kick left *vs.* right	peak >75 d/s identifies poling; slight hump identifies kick	identifies kick left *vs.* right
DS	2 even peaks each cycle (left/right)	cycle visible for some athletes only	2 peaks each cycle, slightly after FwdA	identifies kick left *vs.* right	smaller peak than DP & KDP, <50 d/s	identifies kick left *vs.* right

**Table 2.** Skating technique features common to all athletes.

**Technique**	**FwdA**	**SideA**	**UpA**	**Roll**	**Pitch**	**Yaw**
G2	1 double peak each cycle (for some athletes a minor+major peak)	regular cycle identifies skate left *vs.* right	asymmetric peak preceding FwdA peaks	clear Roll peak >50 d/s follows Yaw peak	peak >50 d/s identifies poling	clear Yaw peak/trough cycle following SideA
G3	2 even peaks each cycle (left/right)	regular cycle identifies skate left *vs.* right	one main peak, between FwdA peaks	irregular peaks <50 d/s	peak >75 d/s identifies poling; 2 Pitch peaks for every Yaw peak	clear Yaw peak/trough cycle following SideA
G4	1 minor peak each cycle (skate) 1 major peak each cycle (skate+poling)	regular cycle identifies skate left *vs.* right	one main peak, coinciding with minor FwdA peak	asymmetrical peaks generally < 50 d/s	peak >75 d/s identifies poling; 1 Pitch peak for every Yaw peak	clear Yaw peak/trough cycle following SideA
G5	2 even peaks each cycle (left/right)	regular cycle identifies skate left *vs.* right	minimal but rhythmic	clear Roll peak >50 d/s follows Yaw peak	peaks <40 d/s much less than G2-G4 (no poling)	clear Yaw peak/trough cycle following SideA
